# The establishment of serum 25-hydroxyvitamin D reference intervals in children aged 0–14 years in Zigong area, China

**DOI:** 10.1186/s40101-021-00265-x

**Published:** 2021-10-06

**Authors:** Jianhong Yu, Xiaoping He, Shengwei Huang

**Affiliations:** 1 Department of Clinical Laboratory, Zigong First People’s Hospital, Zigong, China; 2 Department of Nuclear Medicine, Zigong First People’s Hospital, Zigong, China

**Keywords:** 25-hydroxyvitamin D, Reference interval, Indirect method, Minors, Zigong area

## Abstract

**Objective:**

To establish the reference interval of serum 25-hydroxyvitamin D (25(OH)D) in apparently healthy children in Zigong, China, using an indirect method to provide a basis for proper clinical diagnosis and treatment.

**Methods:**

A total of 1851 apparently healthy children of the Children’s Health Care Department in Zigong First People’s Hospital between January 2016 and December 2020 were included in the study. The Kolmogorov–Smirnov test was used to analyze the data for normality, and the non-normally distributed data were transformed into approximately normal distribution by Blom's normal rank transformation, and the transformed data were excluded from outliers by the quartile spacing method, and the data were stratified and analyzed according to sex, age, and season. The data were stratified according to sex, age, and season, and the area between the 2.5% and 97.5% percentile points was used as the reference interval.

**Results:**

The serum 25(OH)D data were non-normally distributed. The data were normally distributed after Blom’s normality rank transformation, and 92 cases of outliers were excluded from the transformed data according to the interquartile spacing method. The differences in serum 25(OH)D levels between sex were not statistically significant (*P* > 0.05), and there was no need to establish reference intervals based on sex. There was no statistically significant difference in serum 25-hydroxyvitamin D levels between winter and spring, and also no difference between summer and autumn (*P* > 0.05), and the levels were lower in winter-spring than in summer-autumn. Comparison between age groups showed that there was no statistically significant difference in serum 25(OH)D levels between the < 6 months group and the 6 ~ 11 months group, and between the 6 ~ 9 years group and the 10 ~ 14 years group (*P* > 0.05); serum 25(OH)D levels decreased with increasing age. There was an interactive effect of season and age group on 25(OH)D levels, and the corresponding reference intervals were established according to different seasons and age groups. In summer and autumn, the reference intervals of serum 25(OH)D for < 1 year, 1 ~ 2 years, 3 ~ 5 years, and 6 ~  14 years were 39.86 ~ 151.43, 31.54 ~ 131.65, 22.05 ~ 103.75, and 15.36 ~ 85.53 ng/ml and 24.42 ~ 144.20, 31.54 ~ 131.65, 16.80 ~ 165.68, and 15.46 ~ 85.54 ng/ml in winter and spring, respectively.

**Conclusion:**

Reference intervals for serum 25(OH)D in children of different seasons and ages in Zigong, China, were established to provide a reference for clinical disease diagnosis, treatment, and prognosis determination.

Vitamin D is a fat-soluble vitamin, its classical function is to regulate calcium and phosphorus homeostasis and enhance bone development. Various studies have shown that vitamin D deficiency leads to a variety of clinical disorders such as rickets, fractures, diabetes, asthma, and cardiovascular disease [1–3]. Vitamin D exists in the body mainly in the form of vitamin D_2_ and vitamin D_3_, both of which are converted to 25(OH)D in the liver via blood circulation and then to 1,25-hydroxyvitamin D via the kidneys [4]. Among them, 25(OH)D is the main circulating form of vitamin D in the blood and is recognized as a reliable indicator to evaluate the nutritional status of vitamin D in humans. Reference intervals for serum 25(OH)D in minors have been reported in Shanghai [5] and Quanzhou [6], China, but differences in vitamin D levels may exist in different regions with different levels of UV exposure [7]. The recommended vitamin D intake for children aged 0 to 14 years is 10 μg per day (WS/T 578.4–2018 Dietary Nutrient Reference Intakes for Chinese Residents, http://www.nhc.gov.cn/wjw). Vitamin D use in school-age children is lower than in preschoolers [8]. Therefore, the author used the data available in the laboratory database to establish the reference interval of serum 25-hydroxyvitamin D in apparently healthy children in Zigong, China, using an indirect method. The aim was to provide a basis for proper clinical diagnosis and treatment.

## Material and methods

### Research subjects

Serum 25(OH)D test results were collected from 1911 children who were examined in the Children’s Health Care Department of Zigong First People’s Hospital from January 2015 to December 2020, and 26 cases with abnormal specimen status (such as hemolysis, lipemia, and jaundice) and 34 cases with abnormal blood, liver or kidney function results were excluded, and the final number of cases entered into this study was 1851 (1071 males and 780 females). The outliers were removed using the Turkey method. The study was approved by the Ethics Committee of Zigong First People’s Hospital, and written informed consent was obtained from the guardian.

### Samples, instruments, and reagents

Blood samples (2 ml) were collected in all subjects after a fasting state of at least 8 h, placed in a vacuum blood collection tube containing a procoagulant, centrifuged for 10 min at 5 ℃ with a relative centrifugal force of 3072 × *g*, separated the serum and finished the assay within 2 h. The instrument is the CL-6000i fully automated chemiluminescence immunoassay analyzer (Shenzhen Myriad Biomedical Electronics Co., Ltd.), and the test reagents, calibrators, and quality control products are the supporting products of the instrument. Our laboratory uses Westgard multi-rules (1_3S_, 2_2S_, and R_4S_) for internal quality control. The test project participated in the quality evaluation activities organized by the clinical laboratory center of the National Health Commission every year. The results were satisfactory, and the bias was acceptable. The coefficient of variation of the indoor quality control data for 25-hydroxyvitamin D was less than 8.33%, and the bias of the inter-room quality evaluation data was less than 5.0%.

#### Establishment of the reference interval and statistical methods

EXCEL spreadsheet was used to organize the data, and SPSS 26.0 software was used for statistical analysis of the data. Kolmogorov–Smirnov normality test was used to confirm normal distribution for totality of the data, and the non-normally distributed data were transformed into approximately normal distribution by Blom’s normality rank transformation with SPSS 26.0 software and the normality of the transformed data was tested using Kolmogorov–Smirnov test. The outliers were removed using the Turkey method [9]. Data were analyzed by subgroup according to sex, season (spring: March–May; summer: June–August; autumn: September–November; winter: December–February) and age (< 6 months, 6 ~ 11 months, 1 ~ 2 years, 3 ~ 5 years, 6 ~ 9 years, 1014 years), and the Mann–Whitney *U* test was used for comparisons between 2 groups and the Kruskal–Wallis *H* test, one-way test in a general linear model for interaction between two groups, LSD test for two-way comparison, and the establishment of reference intervals when the difference between two groups was not statistically significant, and the reference intervals were expressed by the percentile method (P 2.5 to P 97.5). The difference was considered statistically significant at *P* < 0.05.

## Results

### Normality test and transformation

**Fig. 1:**
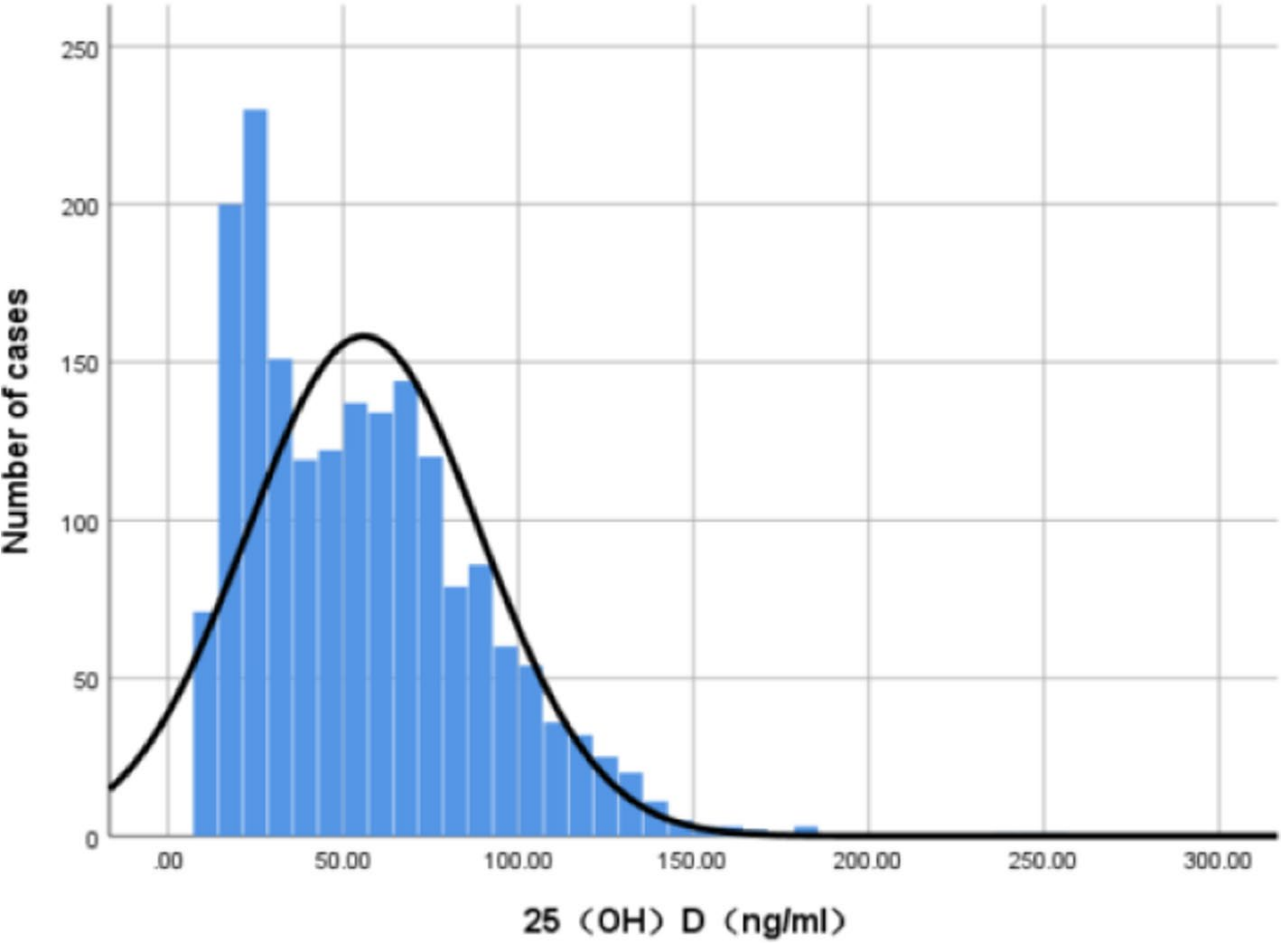
Histogram of distribution of 25(OH)D

**Fig. 2:**
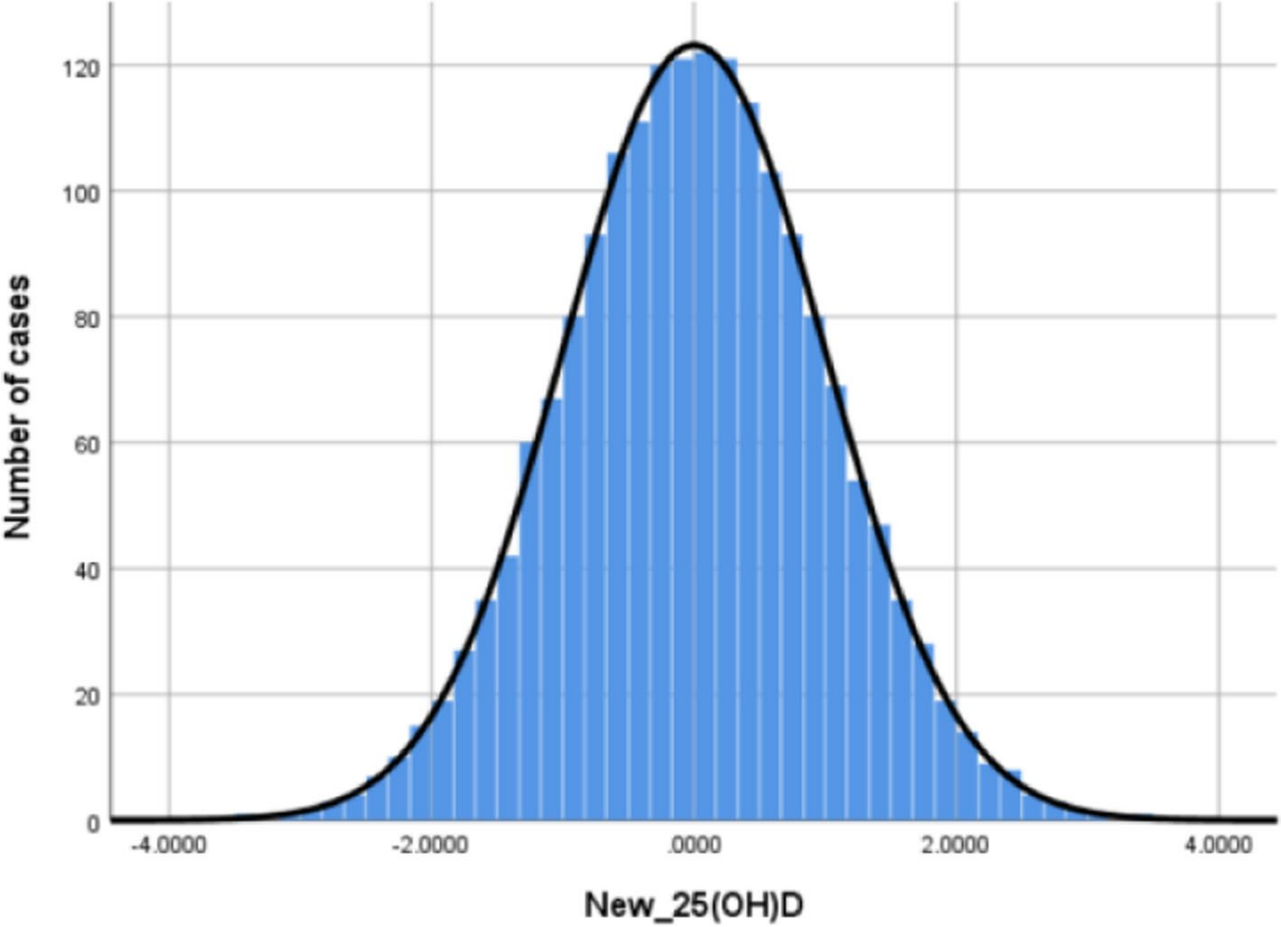
Histogram of distribution of converted 25(OH)D

After the Kolmogorov–Smirnov test, the serum 25-hydroxyvitamin D data showed skewed distribution. Blom’s transformed data showed a normal distribution, as shown in Figs. 1 and 2.

### Outlier rejection

The serum 25-hydroxyvitamin D data were sorted after an Excel spreadsheet. Outliers were removed using the Turkey method, the upper limit is the 75th percentile plus 1.5 times the interquartile range (IQR), and the lower limit is the 25th percentile minus 1.5 times the IQR. Excluding the outliers of 92 cases, the final number of cases entered into the study was 1759.

### Serum 25-hydroxyvitamin D levels between sex

Serum 25-hydroxyvitamin D levels followed a normal statistical distribution in groups < 6 months and 6 ~ 11 months but skewed in groups 1 ~ 2 years, 3 ~ 5 years, 6 ~ 9 years, and 10 ~ 14 years (Kolmogorov–Smirnov test). There was no statistical difference in the composition ratio of sex between the different age groups (*P* > 0.05). There was no statistically significant difference in serum 25-hydroxyvitamin D levels between sex in the same age groups (*P* > 0.05), except for group aged 10 ~ 14 years (Table 1).

**Table 1:** Comparison of serum 25(OH)D levels in the same age groups between sex

Age	Sex	No	25(OH)D (ng/ml)	*P* value
< 6 months	Male	48	78.79 ± 31.12	0.125
	Female	26	90.12 ± 27.75	
6 ~ 11 months	Male	132	71.94 ± 24.07	0.320
	Female	101	72.68 ± 26.56	
1 ~ 2 years	Male	320	68.50 (54.43, 87.18)	0.737
	Female	226	66.80 (54.65, 87.05)	
3 ~ 5 years	Male	223	38.70 (29.50, 56.50)	0.099
	Female	142	44.15 (31.60, 62.60)	
6 ~ 9 years	Male	180	26.90 (21.43,42.23)	0.852
	Female	135	26.40 (22.70, 38.00)	
10 ~ 14 years	Male	120	21.65 (17.53, 31.33)	0.002
	Female	106	25.95 (19.93, 42.03)	

### Serum 25-hydroxyvitamin D levels between seasons

The results of the Kruskal–Wallis H test showed that the differences in serum 25-hydroxyvitamin D levels between different seasons were statistically significant (Table 2). Two-by-two comparison between different seasons using Kruskal–Wallis one-way ANOVA (K-sample) multiple comparison selection all pairwise, listed in Table 3, indicates that there was no statistically significant difference in serum 25-hydroxyvitamin D levels between winter and spring, and also no difference between summer and autumn (*P* > 0.05).

**Table 2:** Comparison of serum 25(OH)D levels between seasons, M (P25 ~ P75)

Season	Number of cases	25(OH)D (ng/ml)
Spring	598	48.10 (26.58 ~ 73.65)
Summer	526	57.25 (33.50 ~ 80.03)
Autumn	347	63.40 (35.90 ~ 85.00)
Winter	288	43.35 (26.43 ~ 71.68)
*Z* value		43.082
*P* value		0.000

**Table 3:** Paired comparison of serum 25(OH)D by season

Season-Season	Test statistics	Standard error	Standard test statistics	*P* value	Adjusted *P* value
Winter-Spring	33.510	36.431	0.920	0.358	1.000
Winter-Summer	149.602	37.232	4.018	0.000	0.000
Winter-Autumn	211.384	40.488	5.221	0.000	0.000
Spring–Summer	-116.092	30.362	-3.824	0.000	0.001
Spring–Autumn	-177.875	34.277	-5.189	0.000	0.000
Summer-Autumn	-61.782	35.127	-1.759	0.079	0.472

### Serum 25-hydroxyvitamin D levels among different age groups

The difference in serum 25-hydroxyvitamin D levels between different age groups was statistically significant by the Kruskal–Wallis H test. By Kruskal–Wallis one-way ANOVA test, the differences in serum 25-hydroxyvitamin D levels between the < 6 months group and the 6 ~ 11 months group and between the 6 ~ 9 years group and the 10 ~ 14 years group were not statistically significant (*P* > 0.05). But the differences were statistically significant between groups < 1 year, 1 ~ 2 years, 3 ~ 5 years, and 6 ~ 14 years (Table 4).

**Table 4:** Comparison of serum 25(OH)D levels between different age groups, M (P25 ~ P75)

Age group	Number of cases	25(OH)D (ng/ml)
< 6 months	74	81.60 (63.88 ~ 101.50)^a^
6 ~ 11 months	233	85.40 (66.10 ~ 109.00)^a^
1 ~ 2 years	546	68.20 (54.50 ~ 87.13)
3 ~ 5 years	365	40.70 (30.50 ~ 59.35)
6 ~ 9 years	315	26.70 (22.30 ~ 41.50)^b^
10 ~ 14 years	226	23.10 (18.70 ~ 32.63)^b^
*Z* value		828.836
*P* value		0.000*

^a^No statistical difference between groups aged < 6 months and 6 ~ 11 months.

^b^No statistical difference between groups aged 6 ~ 9 years and 10 ~ 14 years.

*Statistical difference between age groups overall.

### Reference intervals of serum 25-hydroxyvitamin D in different seasons and age groups

In children aged 9 ~ 14 years, although serum 25-hydroxyvitamin D levels were statistically.

different between the sexes, their *Z* values were lower than Z* values and did not require the establishment of reference intervals based on sex (CLSI EP 28-A3C [10]). The seasons were divided into summer-autumn and winter-spring, and the ages were divided into groups aged < 1 year, 1 ~ 2 years, 3 ~ 5 years, and 6 ~ 14 years. There was an interaction of 25-hydroxyvitamin D between different seasons and ages by general linear model test. Two-two comparisons showed that there was no statistically significant difference in serum 25-hydroxyvitamin D levels between seasons in group aged 1 ~ 2 years (*P* > 0.05), no seasonal grouping was required for reference interval establishment in the 1 ~ 2 years group, and the reference range of serum 25-hydroxyvitamin D in children aged 1 ~ 2 years was 31.54 ~ 131.65 ng/ml. In contrast, seasonal differences existed between the remaining age groups, as shown in Table 5.

**Table 5:** Comparison of serum 25(OH)D levels between seasons in the same age group (ng/ml)

Age group	Summer-Autumn						Winter-Spring						*P* value
	*N*	P2.5	P25	P50	P75	P97.5	N	P2.5	P25	P50	P75	P97.5	
< 1 year	172	39.86	71.18	90.40	114.00	151.43	135	24.42	58.40	80.50	95.50	144.20	0.002
1 ~ 2 years	268	34.70	54.55	68.90	87.20	134.38	278	30.65	54.38	67.40	87.03	131.05	0.523
3 ~ 5 years	169	22.05	32.35	48.30	66.05	103.75	196	16.80	28.13	37.90	52.18	165.68	0.000
6 ~ 14 years	264	15.36	21.85	27.5	55.90	85.53	277	15.46	22.00	27.60	56.10	85.54	0.000

## Discussion

Serum 25-hydroxyvitamin D is an important indicator of vitamin D levels. It is associated with human health status, and 25(OH)D levels are negatively associated with all-cause mortality and stroke risk in elderly Chinese [11, 12]. Higher levels of 25(OH)D are associated with lower overall cancer risk [13]. Higher levels of 25(OH)D are also associated with lower all-cause mortality and cardiovascular disease mortality in patients with diabetes [14]. However, vitamin D insufficiency or deficiency is relatively common in Chinese children and adolescents [15] and postmenopausal women [16]. Vitamin D levels can be increased by sunlight exposure or oral vitamin D supplements. Vitamin D levels can be increased by sunlight exposure or oral vitamin D supplements. However, over-supplementation can also be a health threat, and the literature [17] reports that higher lifetime levels of 25(OH)D are associated with an increased risk of leukoaraiosis. Therefore, proper assessment of serum 25(OH)D levels is of great value for health status, disease diagnosis, and prognostic assessment.

Reference intervals are criteria for assessing health status, disease diagnosis, and prognosis. Most clinical decision-making processes are based on the information provided by laboratory reports. Therefore, providing reliable reference intervals is a fundamental task of clinical laboratories [18]. Currently, the reference intervals used in clinical laboratories are mainly derived from the instructions provided by reagent manufacturers, national clinical test protocols, or literature. However, the inconsistency of measurement results among laboratories is prominent due to different testing systems, different testing methods, different reagent manufacturers, and different geographical regions. Therefore, the reference intervals established by each laboratory according to the local population are more valuable for guiding the diagnosis, disease monitoring, and prognosis of diseases. However, the inconsistency of measurement results among laboratories is prominent due to different testing systems, different testing methods, different reagent manufacturers, and different geographical regions. Therefore, the reference intervals established by each laboratory according to the local population are more valuable for guiding the diagnosis, disease monitoring, and prognosis of diseases.

The establishment of reference intervals by the direct method is recommended by the CLSI EP 28-A3C document [10]. However, the direct method has some disadvantages, such as it requires a lot of human and material resources. Also, children may be limited by ethical restrictions, elderly people may be limited by chronic diseases or medication use. Therefore, establishing reference intervals by the direct method is difficult for most laboratories. With the development of information technology, the indirect establishment of reference intervals through a large amount of data in laboratory information systems is accepted by the EP28-A3C document. In this study, the reference intervals for serum 25(OH)D in children in the Zigong region of China were established by statistical analysis based on the results of serum 25(OH)D tests in apparently healthy children in the last 5 years. Serum 25(OH)D levels in children in the Zigong area did not differ statistically significantly between sex, consistent with Xiao Long-necked [19] and others, and were lower in winter and spring and higher in summer and autumn, due to less sunlight and lower UV exposure and reduced skin synthesis in winter and spring than in summer and autumn. 25-hydroxyvitamin D levels are highest in children under 1 year of age and their levels decrease with increasing age, as also observed in Hangzhou, China [20]. The reason for this may be that children under the age of 1 year are mainly fed on breast milk or formula. Also, this group of children will routinely take vitamin D supplementation by oral means and have better compliance with oral vitamin D. As their age increases, children’s physiological needs for vitamin D gradually increase, but vitamin D supplementation is insufficient. Given the interaction of different seasons and ages on serum 25(OH)D levels, this study established reference intervals for serum 25(OH)D in children of different seasons and age groups (Table 5), and serum 25(OH)D levels were higher in children aged 6 ~ 14 years than in Iranian children [21], which may be due to different ethnic groups and different lifestyle habits. The manufacturer’s reagent instructions (> 30 ng/ml as adequate, < 20 ng/ml as deficient, and 20–30 ng/ml as insufficient) referred to the reference interval in this laboratory, the percentage of 25(OH)D insufficiency or deficiency in apparently healthy children amounted to 25.58%. If the health industry-standard (WS/T 677–2020 Screening method for vitamin D deficiency in human population, http://www.nhc.gov.cn/wjw) is used as the judgment standard, the rate of 25(OH)D insufficiency or deficiency is 7.56%, which is mainly distributed in the population aged 6 ~ 14 years old, and the possible reasons are: (1) children in this age group are mostly primary or junior high school students, with increased academic tasks and reduced outdoor sunlight exposure related; (2) children above 6 years of age have increased physiological needs but reduced or missing oral vitamin D preparations. Therefore, time for outdoor exercise should be ensured for primary and secondary school students, serum 25(OH)D levels should be monitored regularly, and vitamin D preparations should be supplemented promptly for those with insufficient vitamin D levels to ensure that their vitamin D is maintained at adequate levels.

In conclusion, this study established reference intervals for serum 25(OH)D in children of different seasons and ages in Zigong, China. When determining the status of vitamin D levels in children, child health practitioners should combine this reference interval with relevant health industry standards to make comprehensive judgments and correct conditions of vitamin D overdose, deficiency, or lack in a timely manner to promote children's health.

## Data Availability

The datasets supporting the conclusions of this article are included within the article.

## Abbreviation

25(OH)D: 25-Hydroxyvitamin D
